# Cholesterol metabolism: a new molecular switch to control inflammation

**DOI:** 10.1042/CS20201394

**Published:** 2021-06-04

**Authors:** Diana Cardoso, Esperanza Perucha

**Affiliations:** 1 Centre for Inflammation Biology and Cancer Immunology, Department of Inflammation Biology, King’s College London, London, U.K.; 2 Centre for Rheumatic Diseases, King's College London, London, U.K.

**Keywords:** cholesterol, immune system, immunometabolism, immunomodulation, Inflammation, metabolism

## Abstract

The immune system protects the body against harm by inducing inflammation. During the immune response, cells of the immune system get activated, divided and differentiated in order to eliminate the danger signal. This process relies on the metabolic reprogramming of both catabolic and anabolic pathways not only to produce energy in the form of ATP but also to generate metabolites that exert key functions in controlling the response. Equally important to mounting an appropriate effector response is the process of immune resolution, as uncontrolled inflammation is implicated in the pathogenesis of many human diseases, including allergy, chronic inflammation and cancer. In this review, we aim to introduce the reader to the field of cholesterol immunometabolism and discuss how both metabolites arising from the pathway and cholesterol homeostasis are able to impact innate and adaptive immune cells, staging cholesterol homeostasis at the centre of an adequate immune response. We also review evidence that demonstrates the clear impact that cholesterol metabolism has in both the induction and the resolution of the inflammatory response. Finally, we propose that emerging data in this field not only increase our understanding of immunometabolism but also provide new tools for monitoring and intervening in human diseases, where controlling and/or modifying inflammation is desirable.

## Introduction

Cholesterol metabolism is generally associated with an unhealthy diet and atherosclerosis. However, it entitles a much more complicated and fascinating metabolic route, where its metabolites and regulatory circuits have profound effects at the cellular and whole organism level. Among these effects, cholesterol metabolism affects the immune response and the capability of organisms to clear infection and tumour cells, while maintaining homeostasis and health. In this work, we aim to summarise some of the fundamental roles of cholesterol metabolism in controlling the fate of both innate and adaptive immune cells. First, we will briefly introduce both the immune response and basic concepts of cholesterol metabolism. We will then address some of the molecular mechanisms that link immune function with cholesterol metabolites and regulatory elements. We will conclude with some relevant examples of how this knowledge is being harnessed to understand human disease, to develop new therapeutic targets and to repurpose current treatments.

## The immune response: inflammation and resolution

Inflammation is considered the body’s protective response against harm. It is mainly mediated and controlled by the immune system, a complex network of cells and molecules distributed throughout the body. The immune system detects and eliminates danger in the form of pathogens, dead cells or stress signals by eliciting an immune response. In brief, upon recognition of antigen, the immune system activates innate immune cells such as monocytes and neutrophils that respond rapidly to the challenge. They secrete soluble mediators like cytokines, complement molecules and prostaglandins, that drive the inflammatory cascade and promote effector functions such as phagocytosis. Innate immune cells recognise pathogen- or danger-associated molecular patterns through specific receptors, such as toll-like receptors (TLRs) [1]. TLR engagement triggers the maturation of monocytes into activated macrophages that produce pro-inflammatory cytokines such as IL-6, TNFα and type I interferons (IFN α and β), which in turn activate the inflammasome. Inflammasomes are protein complexes that promote the activation of caspase-1, enabling the cleavage of pro-interleukin (IL)-1β and pro-IL-18 into active IL-1β and IL-18 cytokines, and not only are crucial detectors for pathogens but also can sense intracellular abnormalities and initiate inflammation [1].

At the same time, dendritic cells (DCs) will detect, process and transport the antigen to the lymph nodes, where it will be recognised by naïve CD4^+^ T helper (Th) cells through their T-cell receptor (TCR). Once activated, Th cells will differentiate into Th1, Th2 and Th17 subsets according to the signals they receive upon activation. These differentiation programmes are controlled by the master transcription factors T-box expressed in T cells (T-bet), Gata3 and retinoic-acid-receptor-related orphan nuclear receptor gamma (RORγt), respectively. Once committed, CD4^+^ T cells will migrate to the site of infection and orchestrate the immune response. They will enhance macrophage differentiation, B-cell activation and antibody production as well as cytotoxicity effector mechanisms mediated by CD8^+^ T and natural killer (NK) cells. Once the concentration of antigen decreases, the majority of the immune cells will undergo apoptosis, while some will survive to form the memory pool and ready to respond to the next challenge. The generation of immunological memory can be considered as a long-term event arisen from an inflammatory response [2].

As important as driving inflammation to eliminate pathogens is the resolution of the process in order to return to homeostasis. Unresolved inflammation causes tissue damage, chronic inflammation and infection, which contribute to the pathogenesis of many diseases such as cancer, rheumatoid arthritis and asthma. The resolution process targets the inhibition of key inflammatory pathways such as NF-κB activation, the transcription of pro-inflammatory cytokines and the recruitment of cells to the site of inflammation. But it is also an active and tightly regulated process where anti-inflammatory mediators and immune-regulatory pathways and cells play a key role. These include cytokines such as IL-10, lipid metabolites (steroids, eicosanoids) and immune cells, like T regulatory (Treg) and B regulatory (Breg) cells [2,3].

### Immunity and metabolism are interlinked

Immune cells need to react rapidly upon encounter with antigen by dividing very actively and acquiring effector function. To do so, immune cells require nutrients to fulfil the anabolic and catabolic processes driving the effector programme, making the immune response a highly energetically demanding process. Hence, we now know that there is a critical link between immunity and metabolism at the cellular, tissue and whole organism level [4,5]. Quiescent immune cells, like naïve and memory cells, fully oxidise pyruvate (obtained from glucose) or acetyl-CoA (obtained from fatty acids) into the tricarboxylic acid cycle, driving oxidative phosphorylation and maximising ATP generation capacity. In contrast, upon antigenic recognition, activated immune cells undergo metabolic reprograming, switching towards aerobic glycolysis as main source of energy. Lipid metabolism also plays an important part in this metabolic switch, by up-regulating cholesterol and fatty acid biosynthesis, as we will describe in detail in the following sections. Perturbations in lipid metabolism reprogramming, i.e. by using pharmacological blockade of cholesterol biosynthesis with statins, have demonstrated that correct lipid metabolism is a requirement not only to meet biosynthetic demands but also to up-regulate glycolytic and oxidative metabolism [6]. This highlights the key role of cholesterol metabolism in controlling and driving metabolic reprogramming in immune cells.

As both metabolic and immune responses are linked, it is not surprising to observe that dysregulation in these processes are common features of the most prevalent human diseases in First World countries [7]. These include conditions that have chronic inflammation as a feature, either in low grade (such as obesity, type 2 diabetes) or high grade (such as rheumatoid arthritis, inflammatory bowel disease or sepsis). Therefore, immune dysfunction can be targeted by modifying cell metabolism. However, while great progress has been made in the field of immunometabolism describing the molecular and cellular pathways that fuel the immune response, more work is expected to come from the use of this knowledge to manipulate the immune response.

## Cellular cholesterol metabolism: import, biosynthesis and efflux

**Figure 1:**
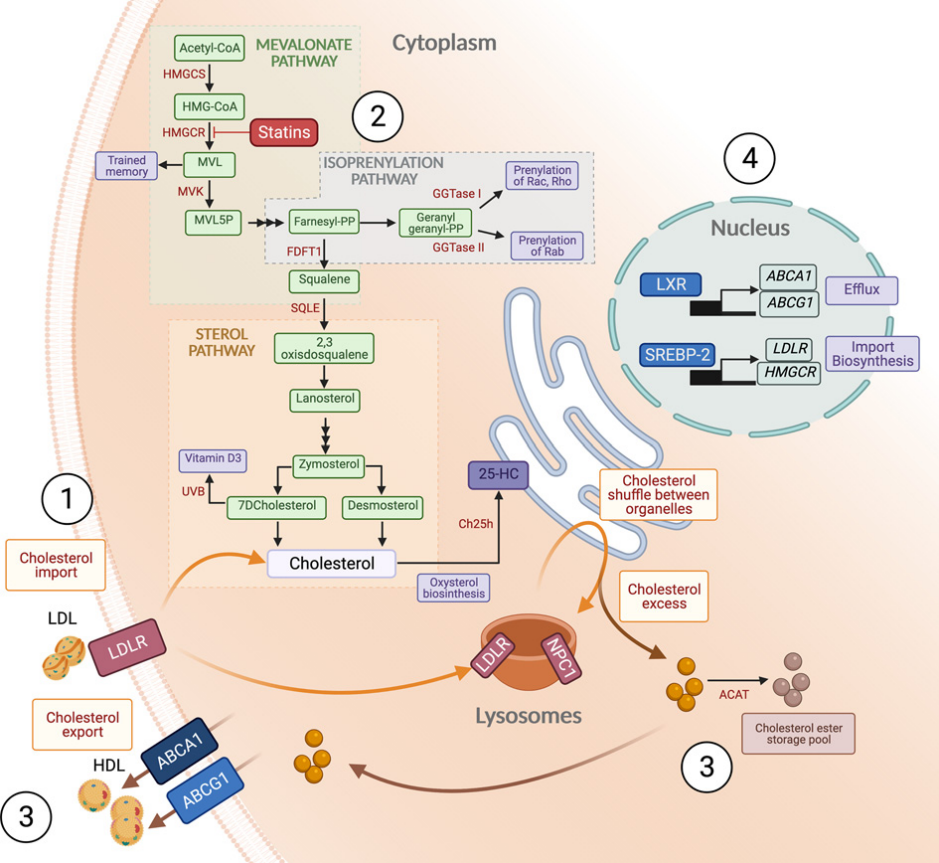
Schematic representation of the cellular cholesterol metabolism 1. Cholesterol is sourced from import of LDL through LDLR; 2. The cholesterol biosynthesis pathway and its branches: the mevalonate pathway, the isoprenylation pathway and the sterol pathway. Some important functions in immune cells are highlighted in purple; 3. Excess cholesterol can be transformed into cholesterol esters or exported through HDL; 4. Cholesterol homeostasis is maintained at the transcriptional level by the actions of SREBP-2 and LXR. Statins inhibit the activity of HMGCR. Abbreviations: 25HC, 25-hydroxycholesterol; ABCA1, ATP-binding cassette transporter 1; ABCG1, ATP-binding cassette subfamily G member 1; ACAT, acyl coenzyme A: cholesterol acyltransferase); Ch25h, cholesterol 25-hydroxylase; FDFT1, farnesyl diphosphate farnesyltransferase 1 or squalene synthase; GGTase, geranylgeranyl transferase; HMG-CoA, 3-hydroxy-3-methylglutaryl-coenzyme A; HMGCS, MHG-CoA synthase; HMGCR, MHG-CoA reductase; LDL, low density lipoproteins;LDLR, LDL receptor; LXR, liver X receptor; MVK, mevalonate kinase; MVL, mevalonate; MVL5P, mevalonate-5-phosphate; NPC1, Nieman-Pick C 1; SQLE, squalene epoxidase; SREBP-2, sensor response element binding protein 2.

Cholesterol is a hydrophobic sterol molecule produced by every nucleated animal cell [8], making up almost 25% of the plasma membrane lipids [9], where it contributes to the control of membrane fluidity and allows for essential functions such as interactions between the cell and pathogens, signal transduction and membrane trafficking [8,10,11]. Unlike other metabolites, cholesterol cannot be catabolised to generate ATP, and it is toxic in excess. Therefore, at the cellular level, cholesterol content has to be tightly monitored and regulated [8]. In this section, we will cover basic concepts of cholesterol metabolism that are important in immune cells, including cholesterol uptake and efflux, the cholesterol biosynthesis pathway as well as the machinery that senses cholesterol inside the cell, driving a tight regulatory circuit (Figure 1).

The majority of cellular cholesterol is imported and distributed from circulating low density lipoprotein (LDL) particles recognised by the LDL receptor (LDLR) and Nieman-Pick C (NPC) proteins 1 and 2 [12]. Myeloid cells can also internalise modified lipoproteins through scavenger receptors like CD36 or lectin-like oxidized low-density lipoprotein receptor-1 [13]. Cholesterol itself can also be synthesised by the cell via the cholesterol biosynthesis pathway (CBP) that provides not only a metabolic route that generates cholesterol but also various metabolites that either directly or indirectly have important functions in immune cells. The CBP can be divided in sub-pathways (Figure 1). The first one, the mevalonate pathway, starts with acetyl-CoA and the subsequent formation of 3-hydroxy-3-methylglutaryl-coenzyme A (HMG-CoA) followed by mevalonic acid synthesis, in a reaction catalysed HMG-CoA reductase (HMGCR), a key regulatory step in the pathway and the target of pharmacological inhibition by statins [14]. Downstream is the formation of farnesyl pyrophosphate (FPP), a metabolite that sits at the junction between the sterol pathway and the isoprenylation pathway. FPP is the precursor of geranylgeranyl pyrophosphate (GGPP) and together they serve as substrates for donation of prenyl groups, via farnesylation and geranylgeranylation, to small GTPases like Rho and Ras families [15]. The prenylation enhances hydrophobicity and facilitates membrane anchoring, which is required for correct positioning and signal transduction. FPP is also the substrate for the synthesis of squalene, which metabolises into 2,3-epoxysqualene via squalene monooxygenase, another crucial regulatory step in the CBP. This step precedes the synthesis of lanosterol, the beginning of the sterol branch of the CBP, which finishes with the synthesis of cholesterol [15,16]. Cholesterol itself is the substrate for the synthesis of important downstream metabolites such as steroid hormones or bile acids, but more relevant in the context of immune cells is the biosynthesis of oxysterols, including 25-hydroxycholesterol (25-HC). 25-HC can be synthesised by the enzyme cholesterol 25-hydroxylase (Ch25h). As the Ch25h-deficient mouse has no metabolic phenotype, the physiological role of 25-HC in systemic cholesterol metabolism is unclear, although its function in regulating the immune response is becoming more apparent and it will be discussed later [17]. Finally, cholesterol generated via the CBP or imported is shuttled between cellular organelles bound to the transporter proteins ATP-binding cassette transporter 1 (ABCA1) and ATP-binding cassette subfamily G member 1 (ABCG1) [18,19]. Nonvesicular transport is also important for cholesterol distribution between cellular structures [20] and to monitor cholesterol content between organelles [21]. Once distributed, excess cholesterol needs to be disposed of to avoid toxicity, either through storage in the form of cholesterol esters through the actions of the acyl coenzyme A: cholesterol acyltransferase (ACAT) enzymes [22] or exported outside the cell [23].

The tight regulation of cholesterol homeostasis occurs through a complex interplay at the transcriptional and post-transcriptional level, allowing fast adaptation to cellular requirements [19] (Figure 1). Intracellular cholesterol is sensed in the endoplasmic reticulum (ER), where levels are regulated through the action of sterol sensors, like sensor response element binding protein 2 (SREBP-2) and the more recently described nuclear erythroid 2 related factor 1 (NRF1) [24]. Under homeostatic conditions, SREBP-2 is kept in the ER linked to SREBP cleavage activating protein (SCAP) and insulin-induced gene 1 protein (INSIG). When cholesterol levels decrease in the ER membrane, the SREBP–SCAP complex migrates from the ER to the Golgi [25]. Here, SREBP-2 is unbound by proteases, allowing its translocation to the nucleus, where it mediates the transcription of key elements of the CBP, including HMGCR and LDLR [8,26]. Of note, SREBP-2 transcription can be activated through the mechanistic target of rapamycin (mTOR) [27], linking two important metabolic sensors in immune cells. On the other hand, excess cholesterol in the ER drives the activation of the liver X receptor (LXR) transcription factors, which mediate cholesterol efflux by controlling the expression of the cholesterol export molecules ABCA1 and ABCG1 [28–31]. The transcriptional function of LXR, a nuclear receptor, is engaged in the presence of increased levels of cholesterol within the cell [32]. How LXR senses ER cholesterol levels is still unclear, as LXRs do not bind cholesterol directly, but they can be activated through oxysterols or sense cholesterol indirectly via NRF1 [24]. Both SREBP-2 and LXR have important functions in controlling the differentiation and effector function of immune cells, sometimes even independently of their role in regulating cholesterol metabolism, as we will discuss in detail.

## Cellular cholesterol immunometabolism

Despite the central role of cholesterol metabolism in cellular biology, our understanding of how it is linked with the innate and adaptive immune responses is just beginning to emerge. Traditionally, the pathway has been associated with cholesterol production within the cell and its role in maintaining membrane fluidity [33] and lipid raft formation. We refer the reader to excellent reviews in this topic [34,35], as in this review we will focus on less well known functions of cholesterol in cells of the immune system. However, cholesterol metabolism also provides metabolites that have specific functions in immune cells, and different than other cells of the body, in a similar way to other metabolic pathways [36]. For instance, we know that signalling downstream antigen recognition (i.e. TLR or TCR) is able to modulate cholesterol metabolism upon activation, whilst cholesterol metabolites can regulate directly or indirectly key immune receptors such as stimulator of interferon response cGAMP interactor (STING) or RORγt. Moreover, systemic immunotherapy with IFN has been known to induce hypercholesterolaemia [37], highlighting the connection between immunity and cholesterol metabolism at both cellular and systemic levels. In this section, we will discuss some of the roles that cholesterol metabolism fulfils in different aspects of the immune response, with a main focus on mechanisms influencing inflammation and resolution.

### Cholesterol immunometabolism in innate immune cells

Pathogens have been shown to harness host cells’ lipid metabolism to fulfil their own metabolic demands [38–40]. As a consequence, host cells down-regulate their lipid metabolism upon microbial sensing to protect the body from infection. Pathogens are sensed by TLR, that together with engagement of type I IFN receptors, have a general outcome to downregulate cholesterol metabolism in order to deplete energy reserves that might be used by the pathogen for propagation [41–45]. Coupled to this response is the regulation of the inflammatory cascade and the macrophage’s effector function, demonstrating the intricate relationship between cholesterol metabolism and the immune response in innate immune cells. Perhaps the best studied example is the formation of foam cells in the atherosclerotic plaque, where excess circulating cholesterol promotes macrophage lipid reprogramming and triggers inflammation. This extensive topic is outside the scope of this review, so we refer the reader to excellent reviews in this field [46,47].

#### Macrophages

Macrophages are at the centre of the innate immune response, and the downstream events involving lipid metabolism and inflammation have been mostly studied in these cells upon TLR4 stimulation. Of note, TLR4 is not only a receptor for endotoxin or certain viral proteins but can also elicit sterile inflammation [48]. Upon TLR4 stimulation and induction of the type I IFN response, both human and mouse macrophages undergo lipid metabolic reprogramming. This includes alterations in sterol content, such as increases in lanosterol and desmosterol content. Lanosterol accumulation, through a still unknown molecular mechanism, interferes with IFNβ gene signature and with signal transducer and activation of transcription (STAT) 1 and STAT2 activation, dampening inflammatory signals and protecting the mice from endotoxemia shock [49]. Moreover, high lanosterol levels improved anti-microbial capacity by increasing membrane fluidity and reactive oxygen species (ROS) production [49]. Similarly, desmosterol accumulation integrates the transcriptional regulation of cholesterol metabolism in foam cells, dampening the inflammatory cascade [50]. Equally important is the requirement of isoprenylation mediators that regulate effector functions such as ROS production, migration and phagocytosis [51] and have been traditionally thought to be pro-inflammatory. Furthermore, inhibition of Geranylgeranyl transferase (GGTase) I has been considered as a therapeutic strategy to treat chronic inflammatory diseases [52]. However, mice with selective GGTase I deficiency in macrophages have increased pro-inflammatory secretion upon stimulation and develop chronic inflammatory arthritis [53]. The reasons behind these conflicting studies are still unknown, but more recent data suggest that GTP-loading of specifically Rac1 (and not other Rho family proteins) is involved in the inflammatory response [54], demonstrating the complexity in the regulation network of these GTPases. Not only metabolites but also metabolic flux through the mevalonate pathway is important. Altered flux perturbs ER cholesterol homeostasis and activates STING, inducing type I IFN gene expression through the phosphorylation of the interferon regulatory transcription factor (IRF) 3 and enhancing the anti-viral effector response [55]. In addition to this, a striking role for mevalonate itself has been described in driving the epigenetic reprogramming required for the acquisition of trained immunity, via activation of insulin-like growth factor 1 receptor (IGFR-1) and mTOR [56,57].

Cholesterol levels in cellular organelles can also regulate macrophage effector response, with excess ER cholesterol content being an activator of NLR family pyrin domain containing (NLRP) 3 inflammasome [58], while in mitochondria it can reduce respiratory capacity, resulting in organelle damage and the release of mitochondrial DNA into the cytosol. This signal activates absent in melanoma 2 (AIM2) inflammasome sensor protein and is sufficient to drive IL-1β processing [44,59].

Another important effect downstream of TLR4 signalling is the regulation of the SREBP-2/LXR axis, with LXR being considered as the key regulator of the cross-talk between lipid sensing and immune function [60]. Initially TLR4 activates mTOR, inducing SREBP-2 processing and transcription of its target genes. Secondly, TLR4 activates type I response and the induction of 25-HC biosynthesis (see below), that overrides mTOR activity and inhibits SREBP2 processing, controlling the overproduction of IL-1β. The generated 25-HC is a natural agonist of LXR, and drives an overall anti-inflammatory programme [60]. In agreement with this model, activation of LXR with pharmacological agonists represses the expression of inflammatory genes by blocking NF-kB activity, inhibiting the production of nitric oxide, cyclooxygenase-2 and IL-6, both *in vitro* and *in vivo* [28,61]. The molecular mechanism of this blockade is still not fully understood, but LXR SUMOylation [62], its role in inducing ABCA1 expression and TLR signalling suppression as a consequence [63] and its capacity to up-regulate IRF8, driving arginase transcription and the anti-inflammatory macrophage programme [64] have all been implicated. On the other hand, LXR signalling deficiency specifically in macrophages shows defects in phagocytosis of apoptotic cells, eliciting a breakdown of self-tolerance [65]. Apoptotic cells are able to activate LXR, delivering a positive cycle that supresses the inflammatory cascade and drives a tolerogenic programme, thus providing another insight into the role of LXR in preventing inflammation and promoting tolerance. Finally, mice deficient in LXR signalling are more susceptible to infection by intracellular pathogens [66], while treatment of mice with LXR agonists can ameliorate inflammatory conditions such as lupus-like autoimmunity [65] and generate protective immune responses against pathogens [67]. Of interest, not all the studies agree with an anti-inflammatory effect of LXR signalling. A recent study reported that LXR agonists, such as T1317 and GW3965, increased synthesis of the pro-inflammatory cytokines IL-6 and IL-8 in human monocytes activated with Bacille Calmette-Guérin, shifting the cells towards a pro-inflammatory phenotype, through metabolic reprogramming in the context of trained immunity [68]. Of note, an LXR-responsive element has been reported in the human TLR4 promoter [69], which might explain some of the pro-inflammatory roles ascribed to LXR activation.

Ch25h catabolises the formation of 25-HC from cholesterol and its expression is tightly regulated due to the potent effects of 25-HC in both cholesterol metabolism and immune function. Ch25h is an interferon induced gene in mouse macrophages and depends on type I (α, β) or II (γ) IFN receptor signalling and downstream STAT1 activation [41,42,70]. Interestingly, Ch25h is not an interferon responsive gene in human cells [71]. The promotor also contains LXR-responsive elements, and LXR agonists induce Ch25h expression, including 25-HC, generating a positive regulatory loop [72]. 25-HC has been demonstrated to have numerous effects in immune cells which have been extensively reviewed elsewhere [17,73–75], so here we will summarise its role in inflammation.

As an LXR endogenous agonist, 25-HC affects immune function by targeting the LXR/SREBP-2 axis as described above [76]. In addition to this, it can exert very potent immunomodulatory roles independently of LXR regulation, via mechanisms that are still poorly understood and somewhat controversial, with both anti- and pro-inflammatory roles described [17,43]. In a landmark study, Reboldi et al*.* observed that upon TLR4 stimulation, Ch25h-deficient macrophages showed increased caspase-1 activity, overexpressing IL-1β and promoting Th17 differentiation as a consequence. Surprisingly, this effect was not LXR, but SREBP pathway dependent [43]. *In vitro*, 25-HC interfered with signalling downstream of TLR4, down-regulating the mRNA levels of the pro-inflammatory cytokines IL-1β, IL-6 and TNFα [77]. Accordingly, Ch25h deficient mice exhibit increased susceptibility to septic shock [43], while 25-HC exerts protective effects in an *in vivo* model of LPS induced-acute lung injury, where systemic levels of pro-inflammatory cytokines were found to be decreased [77]. On the other hand, 25-HC has been shown to amplify inflammatory signalling pathways in both human and mouse macrophages [78,79], and contributing to neuroinflammation by promoting NLRP3 inflammasome assembly, potassium efflux, mitochondria ROS production and LXR activation [80]. Although the mechanisms for this pro-inflammatory role of 25-HC are not understood, it has been recently reported that, by binding directly to surface integrins and activating their downstream signalling, 25-HC can promote the production of inflammatory cytokines [81]. More recently, 25-HC has also been shown to have anti-inflammatory roles, for instance, 25-HC produced by alveolar macrophages promotes lung homeostasis by inducing LXR-dependent resolution of neutrophilia [82]. Finally, it has been shown that 25-HC can be detected in plasma, where normal levels have been quantified as ∼5 ng/ml in humans [70,83] and can significantly increase upon TLR stimulation, to 10 ng/ml in healthy volunteers [70] and up to 200 ng/ml in mouse [84]. However, a possible role of systemic 25-HC has not been investigated to date.

To add another level of complexity, immune signals are able to regulate cholesterol metabolism. Downstream of TLR signalling, NF-κB activation leads to the production of TNFα, a key cytokine regulating inflammation and resolution. Macrophages exposed to TNFα drive the early inflammatory signals required for the initiation of wound healing and the resolution phase of the immune response, while excessive TNFα exposure perpetuates the pro-inflammatory phenotype [85]. These downstream effects of TNFα are partly due to its ability to regulate cholesterol metabolism, by up-regulating ABCA1 expression [86] or in its late phase of exposure (24 h as opposed to 3 h) by activating SREBP-2 activity, that up-regulates not only CBP genes but also the interferon and pro-inflammatory response, thus promoting a pro-inflammatory phenotype, in both human and mouse [87].

#### Dendritic cells

As observed in macrophages, cholesterol accumulation in dendritic cells is sensed as a pro-inflammatory signal. Double Abca1/Abcg1 deficient mice showed enlarged lymph nodes, suggesting a peripheral uncontrolled immune cell proliferation [88]. This defect was attributed partially to enhanced cholesterol accumulation in CD11b^+^ DCs, driving NLRP3 inflammasome activation, secretion of pro-inflammatory cytokines and inducing the expansion of B and T cells [88]. Interestingly, high fat diet can induce this cholesterol accumulation in CD11c^+^ cells, enhancing their capacity to present antigen and promoting autoimmune disease [89]. This report hints at the possibility that diet, or even genetic predisposition to cholesterol accumulation may be factors in the development of autoimmune disease. Oxysterols are also important regulators of DC biology, possibly through their role as endogenous ligands for LXRs. In both human and mouse, they have been shown to promote monocyte differentiation into DCs, inducing the upregulation of molecules required for antigen presentation, migration, and B- and T-cell interaction [90–92], and stimulating T cells to produce pro-inflammatory cytokines [90].

#### Neutrophils

One important defence mechanism against pathogens is the release of neutrophil extracellular traps (NETs), whereby neutrophils enter into a cell death programme (NETosis) that releases chromatin and cellular content into the extracellular environment upon degradation of the cellular membranes. Although the mechanism of NET formation is not fully understood yet, it was demonstrated that treatment with statins or methyl-β-cyclodextrin, whcih deplete membrane cholesterol, promoted the formation of NETs [93,94]. Furthermore, cholesterol crystals and oxidised LDL particles can induce NET formation, together with ROS and IL-1β [95,96]. Oxysterols secreted by the tumour microenvironment have also been shown to regulate neutrophil recruitment in a mechanism dependent on the expression of the chemokine receptor CXCR2 [97]. It would be interesting to explore whether 25-HC has similar effects, as it has been shown that 25-HC levels in sputum correlate with neutrophil counts in chronic obstructive pulmonary disease [98].

#### NK cells

NK cells lay at the interface between innate and adaptive immune responses. Despite their key role in immunity, not much is known about their immunometabolic requirements, especially regarding cholesterol. Assmann et al*.* discovered a new metabolic pathway required for NK cell (and not CD8^+^ T cell) function, driving high levels of glycolysis and oxidative phosphorylation. Surprisingly, this metabolic switch is controlled by SREBP-2 activity [99].

### Cholesterol immunometabolism in adaptive immune cells

#### T cells

Similarly to innate immune cells, antigen recognition, via the TCR in this case, regulates cholesterol metabolism in T cells as part of their metabolic reprograming to fulfil their effector function. As proliferation is a key feature of T-cell effector responses, it is expected that their metabolic reprogramming includes a rapid upregulation of the lipid biosynthetic pathways. Accordingly, it has been known for some time that in lymphocytes, both cholesterol import and active CBP are required for cell cycle progression [100,101]. Cholesterol and cholesterol derivatives shape plasma membrane fluidity and lipid raft dynamics, affecting the formation of the immunological synapse and its downstream signalling events, modulating T-cell activation and function [102,103]. But it was not until 2008 that the relationship between sterol metabolism and T-cell proliferation was described. A landmark study by Bensinger et al. described for the first time the link between immune recognition (TCR signalling) and suppression of the LXR pathway as a requirement to fulfil cellular cholesterol demands for T-cell proliferation [104]. Further studies showed a similar role for SREBP proteins, where SREBP activity not only maintained cellular lipid requirements, but also needed for glycolysis and oxidative phosphorylation metabolic reprogramming [6]. This key finding highlights the interconnection between different metabolic pathways in immune cells, demonstrating the importance of cholesterol metabolism to immune function. At the molecular level, TCR signalling and the downstream activation of phosphatidylinositol-3-kinase, together with mTOR, drives the stabilisation of HMGCR in the ER, promoting the CBP [105].

The role of the CBP in T-cell biology reaches far beyond the control of proliferation and signalling [106], facilitating CD4^+^ and CD8^+^ T-cell differentiation and acquisition of effector function. For instance, naïve CD8^+^ T cells reprogram their cholesterol metabolism upon activation, promoting both import and biosynthesis, while inhibiting efflux [6,104]. Consistent with this observation, inhibition of ACAT1 is able to potentiate CD8^+^ T-cell proliferation and cytotoxic function [107]. Similar findings have been described in naïve CD4^+^ T cells that also up-regulate cholesterol uptake and the CBP, while supressing efflux *in vitro* upon activation [108].

Cholesterol metabolism is also involved in the differentiation fate of CD4^+^ T cells, with increased cholesterol content in the plasma membrane associated with a pro-inflammatory phenotype [109]. Conversely, LXR agonists that will mediate a decrease in plasma membrane cholesterol content have been shown to impair Th1 responses while promoting Treg cell development [31,110], and *in vitro* activation of murine T cells in delipidated media reduced IL-10 production [111]. CD4^+^ T cell fate is affected not only by the cellular cholesterol content but also by metabolites arisen from the pathway. Metabolites of the isoprenylation pathway are integral to the T-cell differentiation programme through their control over the activity of GTPases and cell signalling [112]. The prototype small GTPases, Ras, requires farnesylation for its association with lipid rafts and mediates correct downstream TCR signalling. Accordingly, inhibition of the mevalonate pathway with statins decreases membrane-associated Ras, inhibiting Th1 responses and disease progression in the experimental allergic encephalomyelitis (EAE) mouse model [113]. The provision of GGPP is also important, regulating autophagy, cell size and division in the Jurkat T cell line [114]. More recently, it has been shown that GGPP is a key metabolite in the maintenance of the Treg pool in a mouse model of colitis, amplifying Treg differentiation through STAT5 phosphorylation and decreasing pathogenic Th1 and Th17 responses [45]. The CBP also provides 7-dihydrocholesterol, the precursor of immunomodulatory vitamin D3, which has been directly implicated in the regulation of immune responses via its ability to induce the production of IL-10, a potent anti-inflammatory cytokine [115] and key to the immune-resolution process [116].

The impact of oxysterols has been studied in CD4^+^ T cell biology. The hydroxylated form of 25-HC, 7α,25-HC is the endogenous agonist for Epstein–Barr virus gene 2 (*EBI2*), required for T–B cell interactions in the lymphoid organs, and it will be discussed in detail later. This interaction also promotes the trafficking of CD4^+^ T cells into the central nervous system in the EAE mouse model, highlighting a pro-inflammatory role for oxysterols [117]. But probably the most studied role of oxysterols is in Th17 cell differentiation, a process controlled by the nuclear receptor RORγt [118,119]. Th17 cells are characterised by the production of the pro-inflammatory cytokine IL-17 and have been implicated in the pathogenesis of many inflammatory diseases, tumorigenesis and transplant rejection [120]. Metabolites arisen from the sterol pathway are natural ligands for RORγt [121,122] and are able to boost Th17 differentiation both *in vitro* and *in vivo* [121]. Moreover, Th17 cells accumulate desmosterol to activate RORγt specifically, and this is relevant in the context of Th17 and γδT cell immune responses *in vivo* [108]. These findings suggest that controlling the metabolic flux through the CBP can play an important role not only in Th17 differentiation but also in other processes dependent on RORγt expression, like thymocyte and lymph node development and type 3 innate lymphoid cells. As increased flux through the CBP seems to boost the Th17 differentiation programme, it is not unexpected to find that LXR is a negative regulator of this process [123]. No changes in other cytokines were reported in these studies, but as RORγt has been shown to repress IL-10 in Th17 cells [124], it would be interesting to explore the use of CBP modulation to boost anti-inflammatory responses, for example, in the context of intestinal inflammation.

The role for 25-HC in CD4^+^ T-cell differentiation has generated somewhat contradictory data: Reboldi et al*.* reported in Ch25h-deficient mice increased frequency of Th17 cells, and not Th1 and Th2, due to a higher secretion of IL-1β by the macrophage population [43]. In contrast, 25-HC produced by mouse CD4^+^ T cells inhibited IL-10 expression by reducing the expression of the transcription factor Blimp-1 in an LXR-dependent mechanism [125]. Of note, this report was the first to describe the expression of Ch25h in CD4^+^ T cells and an autocrine role for 25-HC in CD4^+^ T cell differentiation. These data are in agreement with our own work, that described for the first time the effect of 25-HC in human CD4^+^ T cells as a potent inhibitor of IL-10, increasing both Th1 and Th17 responses, possibly via the regulation of c-Maf [126]. Whether these differences are ascribed to different molecular mechanisms, experimental systems or species, they highlight the complexity of the role of cholesterol homeostasis in balancing the pro- and anti-inflammatory programmes in cells of the adaptive immune system.

Together with IL-10 production, Tregs are fundamental to controlling excessive inflammation and keeping the immune response in check. Treg metabolism is heavily reliant on lipid synthesis or uptake to fuel oxidative phosphorylation and generate ATP. Hence, inhibition of glycolysis and mTOR induce Treg differentiation [127]. However, in a really striking report, Zeng et al*.* reported that conditional deletion of mTORC1 in Tregs caused a diminished suppressive function, and the mice developed a systemic inflammatory response as a consequence [128]. The authors observed that the mevalonate pathway was up-regulated downstream of mTORC1 activation and was required for the expression of CTLA-4 and ICOS, key receptors in Treg function [128]. Although the report did not identify the molecular mechanisms linking the mevalonate pathway with Treg function, or whether this effect is also applicable to human Tregs, it highlights the potential side effects of statin treatment. Related to this, LKB1, a metabolic sensor upstream of adenosine monophosphate-activated protein kinase, has been reported to be able to activate the mevalonate pathway, and that this is a requirement for Treg suppressive function and IL-10 expression [129].

Another important point to consider is the effect of systemic cholesterol at the cellular level, both in terms of metabolism and immunity. Although high levels of cholesterol have been traditionally associated with a more pro-inflammatory phenotype, an effect that can be reversed by dietary modification [111], severe hypercholesterolemic mice show an altered Th response, associated with a loss of Th1 IFNγ producing cells and a consequent increase in the frequency of IL-4 and IL-10 producing cells, associated with a Th2 phenotype [130,131]. In the same line, mice fed a high fat diet show decreased mRNA levels of Ch25h, while the altered metabolic parameters can be improved with adenovirus-mediated overexpression of Ch25h [132]. In a humanised mouse model of hypercholesterolaemia, an increased inflammatory response mediated by CD4^+^, but not CD8^+^ T cells, was described [133]. Here, increased numbers of effector CD4^+^ T cells were detected in blood, spleen and lung, while Tregs were reduced [133]. In contrast, hypercholesterolaemia induces Treg differentiation in the liver [134]. In LDLR-deficient mice, that have an activated CBP, the Th17 differentiation programme remains unchanged but intrahepatic Th1 cell differentiation is enhanced. All these data seem to indicate that while intrinsic CBP activity might play a key role in Th1 regulation, exogenous levels of cholesterol, derived from the diet, can instead affect Treg and Th17 production.

#### Cells

Cholesterol metabolism plays critical roles in B-cell biology, although perhaps this has been less studied than in T cells. Functionally, B cells require antigen recognition through the B-cell receptor and CD40 signalling to fulfil their proliferation and differentiation programmes [135]. As such, membrane fluidity controlled by cholesterol content can also alter B-cell antigen presentation and receptor signalling capabilities [136], and membrane cholesterol composition varies at different stages of B-cell activation [137], in a similar way to other cells of the immune system. Moreover, upon CD40 engagement, human B cells up-regulate the CBP, and high concentrations of statin have been shown to down-regulate B-cell activation markers such as CD80, CD86 and HLA-DR [138].

If the CBP is central to B-cell activation and function, it comes as no surprise that several reports have shown an important role for the SREPB-2/LXR regulatory axis in controlling these processes. For instance, in human B cells, LXR synthetic agonist T090137 exerts an inhibitory effect on anti-CD40 and immunoglobulin (Ig) E secretion, reducing allergen-specific IgE in ovalbumin-sensitised mice *in vivo* [139]. At the same time, 25-HC is able to supress B-cell proliferation and IgA immunoglobulin class switching in mice injected with several TLR ligands [84]. However, the molecular connection between 25-HC and CBP regulation has still not been described. More recently, a publication by Trincade et al. have shown that the SREBP-2 pathway is activated downstream of B-cell receptor, but not CD40, signalling. 25-HC via down-regulation of SREBP-2 and cholesterol pathway genes in germinal centre B cells is a requirement for plasma cell differentiation in mice Peyer’s patches [140].

Oxysterols also play an important role in B-cell positioning within lymphoid organs, allowing interaction with follicular dendritic cells and CD4^+^ T helper cells that permits an adequate antibody response. In 2011, two key studies identified the 25-HC hydroxylated form 7α,25-HC as an endogenous agonist for EBI2. EBI2 is an essential component of the humoral response, as it controls the activation and maturation of naive B cells in secondary and tertiary lymphoid tissue [141–143]. 7α,25-HC is produced by the lymphoid stromal cells and establishes a gradient that mediates B-cell migration [17,144], facilitating the interaction between B and T cells, which is required for T-cell dependent antibody responses.

CD40 ligation and TLR9 signals are also the most potent inducers of the regulatory programme in B cells that manifests in the production of IL-10 [145]. Bregs are important to restrict immune responses, and are depleted or functionally impaired in human inflammatory diseases [146,147]. As the CBP is able to regulate CD40 and TLR signals, and correct CBP homeostasis is required for effector T cells to maintain IL-10 expression [126], our group set out to understand the role for cholesterol homeostasis in the regulation of IL-10 in B cells. We discovered that the provision of GGPP via the isoprenylation pathway is able to control phosphatidylinositol-3-kinase signalling and IL-10 expression in TLR9 stimulated human B cells and that this regulation is relevant for controlling Th1 responses *in vitro* [148]. Importantly, this pathway seems to be altered in mevalonate deficient individuals and might contribute to their hyperinflammatory syndrome, as we will discuss later [148].

## Cholesterol immunometabolism and human disease

The CBP plays a crucial role in the pathogenesis of disease with atherosclerosis probably being the most studied example [149,150]. We will now briefly discuss some of the roles that cholesterol metabolism, through regulation of immune responses, has in human disease, with a focus on cancer and inflammatory diseases.

### Anti-tumour immune response and cancer

**Figure 2:**
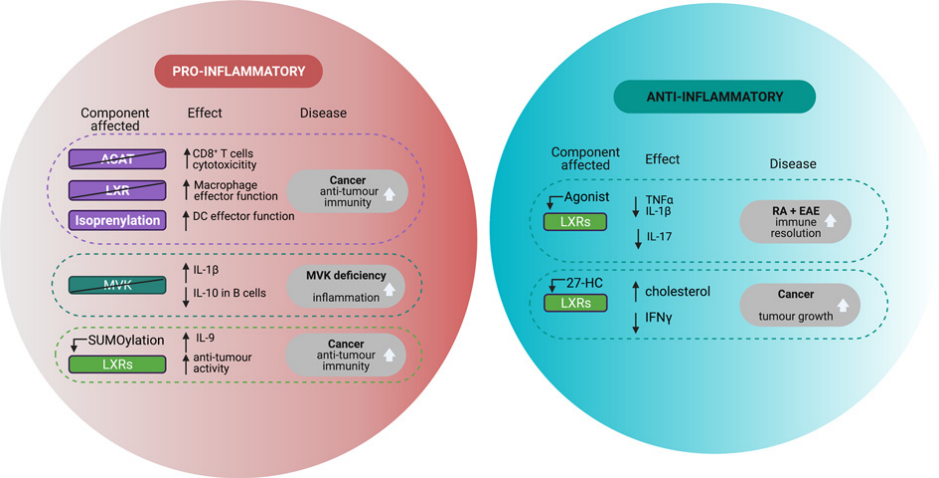
Pro- and anti-inflammatory roles of cholesterol metabolism in disease Manipulating cholesterol metabolism may result in an overall pro- or anti- inflammatory phenotype, which can result beneficial or detrimental outcome in disease. Abbreviations: ACAT, acyl coenzyme A: Cholesterol AcylTransferase); IFNγ, interferon gamma; IL, interleukin; LXR, liver X receptor; MVK, mevalonate kinase; SREBP-2, sensor response element binding protein 2; TNFα, tumour necrosis factor alpha.

Targeting metabolism for therapeutic purposes in the context of cancer is not a new idea [151], but the approaches have been mostly directed to cancer cells. The new knowledge generated by the immunometabolism field provides a fresh perspective, focusing on boosting anti-tumour responses instead [152]. CD8^+^ T cells are central to the anti-tumour response by producing INFγ and granzyme B. Therefore, manipulating cholesterol metabolism to enhance CD8^+^ T cell effector responses can be beneficial in cancer treatment (Figure 2). It has been shown that CD8^+^ T cells with enhanced anti-tumour function have augmented NF-κB pathway activity and IL-9 expression, modulated through SUMOylation of LXR [153]. Consistent with this observation, inhibition of ACAT1 is able to potentiate CD8^+^ T cell proliferation and cytotoxic function [107] and has recently gathered great attention as a potential tool to boost anti-tumour and anti-viral responses. In mice, it was shown that tumours can produce LXR ligands that block expression of CCR7, which prevents DCs undergoing maturation to migrate to lymph nodes around the tumours. Upon injection with tumours with SULT2B1b, which inactivates LXR ligands, migration of DCs and development of inflammation were shown to control tumour growth [154]. ACAT1 inhibitors like Avasimibe are already approved for clinical use and have shown improved outcomes in combination therapy with checkpoint inhibitors in tumour mouse models [107]. Conversely, LXR activation with the oxysterol 27-HC increases levels of cholesterol in macrophages, impairing their ability to promote T-cell expansion and cytotoxic function, with a direct impact on the anti-tumour response [155].

In humans, statin therapy has also gained momentum lately as a beneficial therapy in cancer, as hypercholesterolaemia has been associated with cancer recurrence [156]. Again, most of the studies have focused on the role of statins in depleting cholesterol from cancer cells. However, they might also play a role by boosting the anti-tumour immune response. *In vivo* models have shown a beneficial effect of statin treatment synergising with checkpoint inhibitor blockade in boosting DC antigen presentation and anti-tumour responses by targeting the isoprenylation pathway [157]. In addition to disease models, epidemiological data suggests that statin treatment is associated with reduced cancer mortality [158]. We have shown that statin treatment can boost the effector response of CD4^+^ T cells *in vitro* [126], highlighting a possible dual beneficial role for statins, both depleting cholesterol from tumour cells and improving the effector immune response. However, statins perturb metabolic flux through the mevalonate pathway, impacting isoprenylation, which is also an important pathway involved in the immune response against tumours [159]. In tumour-associated macrophages, the production of pro-inflammatory cytokines is repressed via GGPP interference in TLR signalling, this suggests that TLRs may be an attractive target to induce anti-tumour immunity [160,161].

Tumour cells have also been shown to modify immune cells’ cholesterol metabolism in order to avoid immunosurveillance, and some interesting mechanisms are starting to emerge. Accumulation of mevalonate metabolites in cancer cells is a danger signal which is able to activate immunosurveillance programmes by γδT cells [162] that have key roles in anti-tumour activity [163]. Song et al. described the ability of cancer cells to induce ER stress in CD4^+^ T cells, reducing their capability to produce effector cytokines such as IFNγ, by perturbing N-glycosylation pathways [164]. N-glycosylation requires both glucose import and FPP; although the authors did not explore a role for the CBP, it deserves some consideration. Finally, tumour cells are able to scavenge membrane cholesterol from tumour infiltrating macrophages, inducing a suppressive phenotype [165].

### Genetic defects in cholesterol metabolism: mevalonate kinase deficiency

Mevalonate kinase (MVK) deficiency is an extremely rare autoinflammatory disease characterised by mutations in the *MVK* gene, which results in reduction or absence of mevalonate kinase activity [166]. Patients present with an autoinflammatory phenotype, characterised by recurrent and severe inflammatory episodes and hyperproduction of IL-1β [167] (Figure 2). The blockade in the CBP caused by the MVK deficiency has a double effect. Firstly, it drives accumulation of mevalonate that in macrophages induces trained immunity resulting in greater inflammatory responses [56]. Secondly, there is a defect in the generation of isoprenylation mediators that have been directly implicated in the inability to supress RhoA and the subsequent inflammasome hyperactivation [168]. Deletion of the isoprenylation enzyme PGGT1B supports this, with increased inflammatory cytokine production (including IL-1β) in murine macrophages in response to LPS [160]. Accordingly, therapeutic IL-1β blockade has proven beneficial in the treatment of MVK-deficient patients [169,170].

MVK-deficient patients not only do show an abnormal pro-inflammatory response but also diminished regulatory effector mechanisms. As hyperimmunoglobulinemia is also a key clinical feature, this observation raises the possibility of a defect in B-cell regulation as a contributing factor in the pathology. Accordingly, our research has demonstrated that B cells from MVK-deficient patients are functionally impaired, with a decreased ability to produce IL-10 upon *in vitro* stimulation [148]. Supplementation with GGPP was able to reverse this defect, indicating a defect in isoprenylation as the molecular link between the CBP and IL-10 expression in B cells [148]. Of note, our analyses on the effector function of CD4^+^ T cells in these patients showed no differences when compared to aged and gender matched healthy individuals, highlighting even more the important role of B cells in this disease.

### Chronic inflammation: rheumatoid arthritis

Rheumatoid arthritis (RA) is an autoimmune disorder characterised by a chronic inflammatory phenotype, affecting mainly the joints. It is a debilitating disease, with many associated co-morbidities that impact patients’ daily routines and life expectancy [171]. Due to the inflammatory nature of the disease, it has been postulated that RA patients are at higher risk of cardiovascular disease. However, clinical trials of biological therapy in patients with RA have demonstrated reduced serum cholesterol levels associated with active inflammatory disease. Conversely, serum lipids are increased in patients responding to TNF inhibitors, as the inflammation resolves, in a contradiction that has now been called the ‘lipid paradox’ [172]. This astounding study demonstrates that under conditions of chronic inflammation, hypercholesterolaemia does not increase the risk of cardiovascular disease, as opposed to the general population [172,173]. The mechanism behind these observations is still unclear, but as it is widely accepted that chronic inflammatory diseases arise upon immune homeostasis breakdown, it is conceivable that rather than increasing cardiovascular risk, increased serum cholesterol might impact cellular cholesterol flux that can contribute to immune mechanisms of tolerance. In agreement with this idea, cholesterol homeostasis is required for immune resolution, as our own work has shown [126,148]. In addition to this, in a proof-of-principle small cohort, we have demonstrated that mRNA levels of the cholesterol-25-HC axis consistent with increased inflammatory profile are present in affected tissue (synovial biopsy) of individuals at risk of developing RA, even before the disease manifests [126]. This finding suggests that a metabolic perturbation precedes the breakdown in immune homeostasis, driving chronic inflammation, and chronic inflammatory disease in the long run. Further translational studies are required to clarify this link. Of special interest will be to address the role of systemic cholesterol in determining the fate of the immune response. In this regard, an interesting study has demonstrated that in healthy individuals, statin treatment increased the levels of pro-inflammatory cytokines in serum, although no functional studies in immune cells were performed [174].

*In vivo* models can be useful tools to address some of these questions. In this regard, the use of a LXR agonist has been addressed in the RA *in vivo* model of collagen-induced arthritis, with treated mice showing lower levels of TNFα, IL-1β and IL-6 in serum [168,175] (Figure 2). This finding is also consistent with models of other autoimmune diseases, such the EAE, where LXR agonists have been shown to delay disease development and the differentiation of pathogenic Th17 cells [123,176,177]. However, when similar studies were performed in synovial fluid macrophages isolated from RA patients, the opposite result was found, with LXR activation increasing the secretion of pro-inflammatory IL-6 and TNFα [178]. The already discussed mechanism of LXR pro-inflammatory roles [68,69,179] might explain some of these data. Moreover, these conflicting results between mouse and human data demonstrate the difficulty of understanding the molecular switches that control cholesterol metabolism and their role in immunity, and how much more research is needed to clarify these important questions.

Targeting CBP therapeutically has been proven beneficial in the context of RA. Statins have been shown to exert anti-inflammatory effects through inducing apoptosis in *in vitro* cultured RA synoviocytes [52], while inhibiting the secretion of both pro- and anti-inflammatory cytokines from peripheral blood mononuclear cells of RA patients [180]. Moreover, they have been shown to have a significant anti-inflammatory effect in recent meta-analyses [181]. But on the other hand, statins accelerate the onset of disease in the collagen-induced arthritis model [182] and isoprenylation pathway alterations can induce arthritis in mice, as previously described [53,54]. It is difficult to establish comparisons relating the physiological relevance of statins in human and mouse studies, as lipid metabolism, dosage and drug pharmacology can differ, potentially explaining these contradictory results.

## Concluding remarks

The interlink between metabolism and the immune response is currently a very active area of research. The study of immunometabolism is continually delivering surprising discoveries that change our conception of how immune cells use metabolic enzymes and the metabolites they synthesise. All this knowledge is contributing to a better understanding of the molecular pathways that control the inflammatory response. Indeed, many current drugs used in the clinic to ameliorate a hyperinflammatory state target metabolism; rapamycin or methotrexate being excellent examples. However, cholesterol metabolism has not undergone yet the same momentum than other areas such as glucose metabolism. As we have shown here with many examples, the pro- or anti-inflammatory duality of cholesterol metabolism cannot be applied as a one-size fits all approach. For instance, cholesterol accumulation drives a pro-inflammatory phenotype in innate immune cells, while this has not always been described in adaptive cells, with stark differences between CD4^+^, CD8^+^ and B cells. Besides, there are clear differences in cholesterol metabolism at the species level that might limit the extrapolation of mouse models into human disease [183].

Understanding cholesterol immunometabolism is complicated. The CBP provides substrates for multiple cellular and immunological processes that are deeply interlinked with other metabolic programmes and key metabolic regulators of the cell, such as aerobic glycolysis and mTOR, as we have described here, making it sometimes difficult to ascertain precisely what pathway or metabolite regulate a specific molecular process. In our opinion, this is partly due to the lack of widely accessible, reliable and simplified methods to measure the CBP and its metabolic flux, in a similar way that the Seahorse extracellular flux technology provides readouts for glycolysis and oxidative phosphorylation.

We believe that further understanding of the molecular events that link cholesterol metabolism, cellular sensing and immune effector molecules will be key to deliver improvements in the field. This should be accompanied by the development of tools and techniques that allow a reliable and easy measurement of metabolites and metabolic flux. Finally, studies should also be aimed at understanding the relationship between systemic and cellular cross-talk, with special emphasis on disease states.

## Open Access

Open access for this article was enabled by the participation of King’s College London in an all-inclusive *Read & Publish* pilot with Portland Press and the Biochemical Society under a transformative agreement with JISC.

## Abbreviations

ABCA1: ATP-binding cassette transporter 1
ABCG1: ATP-binding cassette subfamily G member 1
ACAT: acyl coenzyme A: cholesterol acyltransferase
AIM2: absent in melanoma 2
Breg: B-regulatory cell
CBP: cholesterol biosynthesis pathway
Ch25h: cholesterol 25-hydroxylase
DC: dendritic cell
EAE: experimental allergic encephalomyelitis
EBI2: Epstein-Barr virus gene 2
ER: endoplasmic reticulum
FPP: farnesyl pyrophosphate
GGPP: geranylgenranyl pyrophosphate
GGTase: geranylgeranyl transferase
HC: hydroxycholesterol
HMG-CoA: 3-hydroxy-3-methylglutaryl-coenzyme A
HMGCR: MHG-CoA reductase
IFN: interferon
Ig: immunoglobulin
IGFR-1: insulin-like growth factor 1 receptor
IL: interleukin
INSIG: insulin-induced gene 1 protein
IRF: interferon regulatory transcription factor
LDL: low density lipoprotein
LDLR: LDL receptor
LXR: liver X receptor
mTOR: mechanistic target of rapamycin
MVK: mevalonate kinase
NETs: neutrophil extracellular traps
NK: natural killer
NLRP3: NLR family pyrin domain containing 3
NPC: Nieman-Pick C
NRF1: nuclear erythroid 2 related factor 1
RORγt: retinoic-acid-receptor-related orphan nuclear receptor gamma
ROS: reactive oxygen species
SCAP: SREBP cleavage activating protein
SREBP-2: sensor response element binding protein 2
STAT: signal transducer and activation of transcription
STING: stimulator of interferon response CGAMP interactor
T-bet: T-box expressed in T cells
TCR: T cell receptor
Th: T-helper cell
TLR: Toll-like receptor
Treg: T-regulatory cell
